# Oncofinder, a new method for the analysis of intracellular signaling pathway activation using transcriptomic data

**DOI:** 10.3389/fgene.2014.00055

**Published:** 2014-03-25

**Authors:** Anton A. Buzdin, Alex A. Zhavoronkov, Mikhail B. Korzinkin, Larisa S. Venkova, Alexander A. Zenin, Philip Yu. Smirnov, Nikolay M. Borisov

**Affiliations:** ^1^Pathway Pharmaceuticals, LimitedWan Chai, Hong Kong, Hong Kong; ^2^D. Rogachev Federal Research Center of Pediatric Hematology, Oncology and ImmunologyMoscow, Russia; ^3^Shemyakin-Ovchinnikov Institute of Bioorganic ChemistryMoscow, Russia; ^4^Biological and Medical Physics, Moscow Institute of Physics and TechnologyDolgoprudny, Russia; ^5^Burnasyan Federal Medical Biophysical CenterMoscow, Russia

**Keywords:** mitogenic signaling pathways, microchip transcriptome investigation, targeted anti-cancer drugs, expression level profiling, signalome profiling, stochastic robustness analysis

## Abstract

We propose a new biomathematical method, OncoFinder, for both quantitative and qualitative analysis of the intracellular signaling pathway activation (SPA). This method is universal and may be used for the analysis of any physiological, stress, malignancy and other perturbed conditions at the molecular level. In contrast to the other existing techniques for aggregation and generalization of the gene expression data for individual samples, we suggest to distinguish the positive/activator and negative/repressor role of every gene product in each pathway. We show that the relative importance of each gene product in a pathway can be assessed using kinetic models for “low-level” protein interactions. Although the importance factors for the pathway members cannot be so far established for most of the signaling pathways due to the lack of the required experimental data, we showed that ignoring these factors can be sometimes acceptable and that the simplified formula for SPA evaluation may be applied for many cases. We hope that due to its universal applicability, the method OncoFinder will be widely used by the researcher community.

Intracellular signaling pathways (SPs) regulate numerous processes involved in normal and pathological conditions including development, growth, aging, and cancer. Many bioinformatic tools have been developed recently that analyze SPs. However, none of them makes it possible to efficiently do the high-throughput quantification of pathway activation scores for the individual biological samples. Here we propose a method for quick, informative and large-scale screening of changes in signaling pathway activation (SPA) in cells and tissues. These changes may reflect various differential conditions like differences in physiological state, aging, disease, treatment with drugs, infections, media composition, additives, etc. One of the potential applications of SPA studies may be in utilizing mathematical algorithms to identify and rank the medicines based on their predicted efficacy.

The information about SPA can be obtained from the massive proteomic or transcriptomic data. Although the proteomic level may be somewhat closer to the biological function of SPA, the transcriptomic level of studies today is far more feasible in terms of performing experimental tests and analyzing the data. The transcriptomic methods like Next-generation sequencing (NGS) or microarray analysis of RNA can routinely determine expression levels for all or virtually all human genes (Shirane et al., 2004). Transcriptome profiling may be performed for the minute amount of the tissue sample, not necessarily fresh, but also for the clinical formalin-fixed, paraffin-embedded (FFPE) tissue blocks. For the molecular analysis of cancer, gene expression can be interpreted in terms of abnormal SPA features of various pro- and antimitotic SPs. Such analysis may improve further decision-making process of treatment strategy selection by the clinician.

Pro- and antimitotic SPs that determine various stages of cell cycle progression remained in the spotlight of the computational biologists for more than a decade (Kholodenko et al., 1999; Borisov et al., 2009; Kuzmina and Borisov, 2011). Today, hundreds of SPs and related gene product interaction maps that show sophisticated relationships between the individual molecules, are cataloged in various databases like UniProt (The UniProt consortium, 2011), HPRD (Mathivanan et al., 2006), QIAGEN SABiosciences (SABiosciences), WikiPathways (Bauer-Mehren et al., 2009), Ariadne Pathway Studio (Nikitin et al., 2003), SPIKE (Elkon et al., 2008), Reactome (Haw and Stein, 2012), KEGG (Nakaya et al., 2013), etc. One group of bioinformatic approaches integrated the analysis of transcriptome-wide data with the models employing the mass action law and Michaelis-Meten kinetics (Yizhak et al., 2013). These methods which were developing during last 15 years, however, remained purely fundamental until recently, primarily, because of the multiplicity of interaction domains in the signal transducer proteins that enormously increase the interactome complexity (Conzelmann et al., 2006; Borisov et al., 2008). Secondly, a considerable number of unknown free parameters, such as kinetics constants and/or concentrations of protein molecules, significantly complicated the SPA analysis. Yizhak et al. (2013) suggested that the clinical efficiency of several drugs, e.g., geroprotectors, may be evaluated as the ability to induce the kinetic models of the pathways into the steady state. However, protein-protein interactions were quantitatively characterized in detail only for a tiny fraction of SPs. This approach is also time-consuming since to process each transcriptomic dataset it requires extensive calculations for the kinetic models (Yizhak et al., 2013).

However, all the contemporary bioinformatical methods that were proposed for digesting large-scale gene expression data followed by recognition and analysis of SPs, have an important disadvantage. They do not allow tracing the overall pathway activation signatures and quantitively estimate the extent of SPA (Kuzmina and Borisov, 2011; Hwang, 2012; Yizhak et al., 2013). This may be due to lack of the definition of the specific roles of the individual gene products in the overall signal transduction process, incorporated in the calculation matrix used to estimate SPA.

Here we propose a new method that, to our knowledge, for the first time makes it possible to quantitatively estimate SPA for individual samples basing on the large-scale gene expression data. The method was previously announced by our team here (Zhavoronkov et al., 2014). Theoretically, the signal transduction efficiency at every stage of the SP depends on the concentrations of the interacting gene products. The computational modeling of the signal transduction processes indicated that most of the interacting proteins can be found in the living cells at the concentrations significantly lower than the saturation levels for each transduction step (Birtwistle et al., 2007; Borisov et al., 2009). Our model is based on the correlation of the signal transducer concentrations and the overall SPA. We also determined the overall individual roles of certain gene products in the functioning of each individual SP. These roles can be either positive or negative signal transduction regulators; alternatively, for some proteins the roles may be undefined or neutral. Finally, these roles may be characterized quantitatively depending on the individual importances of the individual interactors in the overall SPA. The determination of these roles for each individual SP is a non-trivial task that has several uncertainties. Namely, protein interactions within each pathway may be competitive or independent, and therefore, belong to a sequential or parallel series of the nearby events (Borisov et al., 2006; Conzelmann et al., 2006). The overall graph for the protein interaction events may include both sequential (pathway-like) and parallel (network-like) edges (Conzelmann et al., 2006; Borisov et al., 2008). The role of each gene product in the signal transduction may depend on whether it works in a sequential or a parallel way. Alternatively, as the raw approximation of this situation, one may propose a simplified method that utilizes only the overall roles of each gene product in the SPA. In this case, each simplified signaling graph includes only two types of branches of protein interaction chain: one for sequential events that promote SPA, and another for repressor sequential events. Under these conditions, it can be presumed that all activator/repressor members have equal importances for the SPA, and come to the following formula for the overall signal outcome (*SO*) of a given pathway, $SO=\frac{\prod_{i\;=\;1}^{N}{\left[AGEL\right]}_{i}}{\prod_{j\;=\;1}^{M}{\left[RGEL\right]}_{j}}$. Here the multiplication is done over all possible activator and repressor proteins in the pathway, [*AGEL*]_*i*_ and [*RGEL*]_*j*_ are relative gene expression levels of activator (*i*) and repressor (*j*) members, respectively. To obtain an additive value, it is possible to take the logarithmic levels of gene expression, and thus come to a function of *pathway activation strength*, *PAS*, which operates with the experimental datasets obtained during comprehensive profiling of gene expression, for a pathway *p*, *PAS_p_* = ∑*_n_ARR_np_* · lg(*CNR_n_*). Here the *case-to-norm ratio*, *CNR_n_*, is the ratio of the expression levels of a gene *n* in the sample (e.g., of a cancer patient) and in the control (e.g., average value for healthy group). The discrete value *ARR* (*activator*/*repressor role*) shows whether the gene product promotes SPA (1), inhibits it (−1) or plays an intermediate role (0.5, 0 or −0.5, respectively). Negative and positive overall *PAS* values correspond, respectively, to decreased or increased activity of SP in a sample, with the extent of this activity proportional to the absolute value of *PAS*.

However, the assumption of sequential protein-protein interaction in pathways may seem rather artificial. Although it is difficult to precisely estimate the importance of certain gene products that act in the pathway in a non-sequential mode, the solution may come from the kinetic models of SPA that use the “low-level” approach of mass action law describing each act of protein interactions. Some of these models were previously experimentally validated by us and others using Western blot analysis (Kholodenko et al., 1999; Kiyatkin et al., 2006; Birtwistle et al., 2007; Borisov et al., 2009; Kuzmina and Borisov, 2011). Our previous experience suggests that the two approaches can be used to estimate the importance of distinct genes/proteins in the pathways. One of them operates with the concept of sensitivity of the ordinary differential equation system with the free parameters (Kholodenko et al., 2003), which is generally applied to kinetic constants, but may be used for assperating with the protein concentrations in the kinetic model of a pathway (Kuzmina and Borisov, 2011), according to a formula,$w_{j}^{\left(1\right)}=\frac{1}{T}\int_{0}^{T}\left|\frac{\partial \text{ln}[EFF(t)]}{\partial {\text{ln}}_{j}C^{\;tot}}\right|dt$. Here *w* is the importance factor, [*EFF*(*t*)] is the time-dependent concentration of the active pathway effector protein (experimentally traced marker of a pathway activation), the upper integration limit *T* is the time of reaching the steady-state, and *C^tot^_j_* is the total concentration for the protein *j*.

Another way to calculate the importance factor for the gene products deals with the stiffness/sloppiness analysis of the effector activation (Daniels et al., 2008). This approach comprises analyzing the Hesse matrix, $H_{ij}=\frac{\partial^{2}}{\partial C_{i}^{\;tot}\partial C_{j}^{\;tot}}\text{​}\sum_{k}\frac{{\left(\left[EFF\left(C^{\;tot},t_{k}\right)\right]-{\left[EFF\right]}_{k}^{\exp}\right)}^{2}}{\sigma_{k}^{2}}$, where **C***^tot^* is the vector of total concentrations for every protein in the pathway, [EFF (*C^tot^*, *t_k_*)] is concentration of an active pathway effector protein at the time point *t_k_*, [*EFF*]^exp^_*k*_ is the experimentally measured (e.g., by Western blots) total concentration of the effector at the same time, and σ_*k*_ is the experimental error for this measurement. The sloppiness/stiffness analysis looks for the eigenvalues, λ_*m*_, and eigenvectors, **ξ**_*m*_, for the Hesse matrix, **Hξ**_*m*_ = λ_*m*_ · **ξ**_*m*_. The higher is the absolute value of λ_*n*_, the “stiffer” is the direction within the *n*-dimensional space of **C**^*tot*^ (where *n* is the number of protein types in the pathway model). The eigenvector components along with the stiffest direction, **ξ**_*s*_, may be used for assessment of the importance factor *w* of a certain gene products in a pathway according to the formula: *w*^(2)^_*j*_ = |ξ_*sj*_|.

Taking into account the above considerations, we come to the following final formula for assessing the SPA: *PAS*^(1, 2)^_*p*_ = ∑*_n_ARR_np_* · *BTIF_n_* · *w*^(1,2)^_*n*_ · lg (*CNR_n_*). Here the Boolean flag *BTIF* (*beyond tolerance interval flag*) indicates that the expression level for the gene n for the given sample is different enough from the respective expression level in the reference sample or set of reference samples. For this demonstration of our method we applied two simultaneous restriction/inclusion criteria to the expression of each individual gene: (i) 50% expression level cut-off rate compared to the average for the reference set, and (ii) the sample expression level should differ stronger than two standard deviations from the average of the reference set.

We next explored the effect of the introduction of the importance factors *w* in calculating *PAS* compared to the simplified model of *PAS* evaluation lacking *w*. Importance factors were calculated using either sensitivity-based, *w*^(1)^, or stiffness-based, *w*^(2)^, algorithms. We performed this verification for the EGFR pathway, for which we established and published this model previously (Kuzmina and Borisov, 2011). For these two sets of the importance factors, and for the *w*-free model, we performed a computational analysis of nine transcriptomes established using microarray hybridization technology for human glioblastoma samples from the published datasets (Supplementary dataset 1). The information on SP organization was taken from the Web-based SABiosciences database. The data on *ARR* were manually curated by analyzing the same database. Our findings suggest that the cloud of values for the ratio $\frac{PAS_{\text{EGFR}}^{\left(1\right)}}{PAS_{\text{EGFR}}}$ (where *PAS*_EGFR_ is the *PAS* value for the EGFR pathway in the simplified model, where all importance factors equal to 1) lies within the interval of (0.6 ± 0.8), whereas the ratio $\frac{PAS_{\text{EGFR}}^{\left(2\right)}}{PAS_{\text{EGFR}}}$ belonged to the interval (1.0 ± 0.8). Overall, we conclude that for such a complex SP like EGFR which includes >300 gene products, incorporation of the importance factors had only a moderate effect on the *PAS*. This suggests that, in principle, the simplified formula for *PAS* calculation may be applied for the pathway analysis.

For the overwhelming majority of the SPs, there is no experimental data available that makes impossible for them to calculate the importance factors using kinetic models. For them we performed the stochastic robustness analysis using the simplified formula for *PAS*. We introduced the additional random perturbation factor, *w_n_*, which was used as the analog of importance factor for *PAS* evaluation. In our computational simulation, the distribution of *w_n_* was logarithmically normal and calculated as follows: *w_n_* = 2*^x_n_^*, where *x_n_* were normally distributed random numbers with the expected value of *M* = 0 and standard deviation σ = 0.5. The random perturbation factors *w_n_* were applied to the glioblastoma transcriptional dataset GSM215422 (GSM215422 dataset). Importantly, although the perturbation was done independently 98 times with independent weighting factors *w_n_*, for each gene, the values of standard deviation for the set of alternate *PAS* (*APAS*) were nor big enough to mask the proportional trend between the average perturbed *PAS* and unperturbed *PAS* for each of the 68 SPs analyzed in this study (Figure 1; Supplementary dataset 2).

**Figure 1:**
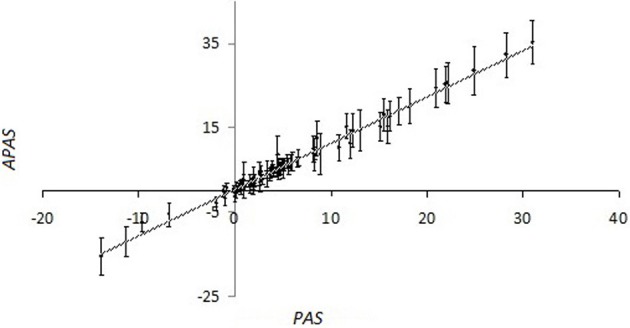
**Values of pathway activation strength (*APAS*) that were calculated using the 98 random trials, each having random log-normally distributed weighting factors *w_n_*, vs. non-perturbed *PAS* for the different SPs, calculated using OncoFinder method**. The pathway information was extracted from the SABiosciences database. Primary data are shown on the Supplementary dataset 3. For the perturbed values (*APAS*), both average values (points at the plot) and standard deviation bars are shown.

We propose here a new biomathematical method, OncoFinder, for both quantitative and qualitative analysis of the intracellular SP activation. It can be used for the analysis of any physiological, stress, malignancy and other perturbed conditions at the molecular level. The enclosed mathematical algorithm enables processing of high-throughput transcriptomic data, but there is no technical limitation to apply OncoFinder to the proteomic datasets as well, when the developments in proteomics allow generating proteome-wide expression datasets. We hope that due to its universal applicability, the method OncoFinder will be widely used by the biomedical researcher community and by all those interested in thorough characterization of the molecular events in the living cells. We also want to encourage building international scientific partnership aimed at the standardized experimental characterization of the importance factors for individual proteins, starting at least with the SPs most relevant to the major aspects of human physiology.

## Conflict of interest statement

The authors declare that the research was conducted in the absence of any commercial or financial relationships that could be construed as a potential conflict of interest.
