# Supplementary material for: Oncofinder, a new method for the analysis of intracellular signaling pathway activation using transcriptomic data
Source: Front Genet. 2014 Mar 25;5:55. doi: 10.3389/fgene.2014.00055 (PMC3971199; doi:10.3389/fgene.2014.00055)
Supplement: Supplementary file 1 [file DataSheet1.DOC]

Supplementary Dataset 1. PAS values calculated for EGFR signaling pathway using different models of evaluating importance factor *w* for individual proteins.

| Sample | PAS_EGFR | PAS^1_EGFR | PAS^2_EGFR | n^1 = PAS^1_EGFR/PAS_EGFR | n^2 = PAS^2_EGFR/PAS_EGFR |
| --- | --- | --- | --- | --- | --- |
| GSM215418 | 13,53 | 9,99 | 19,04 | 0,738359202 | 1,407243163 |
| GSM215420 | 6 | -1,41 | 4,01 | -0,235 | 0,668333333 |
| GSM215422 | 20,14 | 10,04 | 21,92 | 0,498510427 | 1,088381331 |
| GSM215423 | -3,77 | -3,46 | -8,05 | 0,917771883 | 2,135278515 |
| GSM215425 | 6,2 | 3,31 | 9,3 | 0,533870968 | 1,5 |
| GSM215426 | 5,23 | 10,55 | 3,84 | 2,017208413 | 0,734225621 |
| GSM215427 | 5,47 | -4,62 | -2,8 | -0,844606947 | -0,511882998 |
| GSM215428 | 13,75 | 14,27 | 19,74 | 1,037818182 | 1,435636364 |
| GSM215429 | 7,07 | 4,45 | 1,94 | 0,629420085 | 0,274398868 |
|  |  |  |  | Average | Average |
|  |  |  |  | 0,588150246 | 0,970179355 |
|  |  |  |  | Standard deviation | Standard deviation |
|  |  |  |  | 0,799896547 | 0,781602294 |
