# Supplementary material for: Oncofinder, a new method for the analysis of intracellular signaling pathway activation using transcriptomic data
Source: Front Genet. 2014 Mar 25;5:55. doi: 10.3389/fgene.2014.00055 (PMC3971199; doi:10.3389/fgene.2014.00055)
Supplement: Supplementary file 2 [file DataSheet2.DOC]

Supplementary Dataset 2.

List of the SPs used for evaluation of the PAS robustness analysis.

| **Pathway name** | **Link to the Pathway** |
| --- | --- |
| Caspase_Cascade | http://www.qiagen.com/products/genes%20and%20pathways/Pathway%20Details.aspx?pwid=84& |
| DNA-damage-induced_apoptosis_effect | http://www.qiagen.com/products/genes%20and%20pathways/Pathway%20Details.aspx?pwid=130& |
| DNA-damage-induced_responses_effect | http://www.qiagen.com/products/genes%20and%20pathways/Pathway%20Details.aspx?pwid=137& |
| DNA_Repair_Mechanisms_Pathway | http://www.qiagen.com/products/genes%20and%20pathways/Pathway%20Details.aspx?pwid=137& |
| Hedgehog _Pathway | http://www.qiagen.com/products/genes%20and%20pathways/Pathway%20Details.aspx?pwid=220& |
| Mitochondrial_Apopotosis_Pathway | http://www.qiagen.com/products/genes%20and%20pathways/Pathway%20Details.aspx?pwid=295& |
| NGF_m_Pathway | http://www.qiagen.com/products/genes%20and%20pathways/Pathway%20Details.aspx?pwid=320& |
| Notch_Pathway | http://www.qiagen.com/products/genes%20and%20pathways/Pathway%20Details.aspx?pwid=325& |
| p53_Signaling_m_Pathway | http://www.qiagen.com/products/genes%20and%20pathways/Pathway%20Details.aspx?pwid=339& |
| PTEN_Pathway | http://www.qiagen.com/products/genes%20and%20pathways/Pathway%20Details.aspx?pwid=375& |
| SMAD_Pathway | http://www.qiagen.com/products/genes%20and%20pathways/Pathway%20Details.aspx?pwid=412& |
| TNF_m_Pathway | http://www.qiagen.com/products/genes%20and%20pathways/Pathway%20Details.aspx?pwid=437& |
| TRAF_m_Pathway | http://www.qiagen.com/products/genes%20and%20pathways/Pathway%20Details.aspx?pwid=448& |
| ATM_Pathway | http://www.qiagen.com/products/genes%20and%20pathways/Pathway%20Details.aspx?pwid=46& |
| Glucocorticoid_Receptor_Pathway | http://www.qiagen.com/products/genes%20and%20pathways/Pathway%20Details.aspx?pwid=205& |
| AHR_Pathway | http://www.qiagen.com/products/genes%20and%20pathways/Pathway%20Details.aspx?pwid=19& |
| BRCA1_Pathway | http://www.qiagen.com/products/genes%20and%20pathways/Pathway%20Details.aspx?pwid=46& |
| Chemokine_Pathway | http://www.qiagen.com/products/genes%20and%20pathways/Pathway%20Details.aspx?pwid=106& |
| Chromatin_Remodeling_Pathway | http://www.qiagen.com/products/genes%20and%20pathways/Pathway%20Details.aspx?pwid=110& |
| Circadian_Pathway | http://www.qiagen.com/products/genes%20and%20pathways/Pathway%20Details.aspx?pwid=113& |
| Fas_m_Signaling_Pathway | http://www.qiagen.com/products/genes%20and%20pathways/Pathway%20Details.aspx?pwid=175& |
| GPCR_Pathway | http://www.qiagen.com/products/genes%20and%20pathways/Pathway%20Details.aspx?pwid=207& |
| Interferon_Pathway | http://www.qiagen.com/products/genes%20and%20pathways/Pathway%20Details.aspx?pwid=255& |
| JNK_Pathway | http://www.qiagen.com/products/genes%20and%20pathways/Pathway%20Details.aspx?pwid=266& |
| Mismatch_repair_effect | http://www.qiagen.com/products/genes%20and%20pathways/Pathway%20Details.aspx?pwid=293& |
| p38_Signaling_Pathway | http://www.qiagen.com/products/genes%20and%20pathways/Pathway%20Details.aspx?pwid=337& |
| PAK_Pathway | http://www.qiagen.com/products/genes%20and%20pathways/Pathway%20Details.aspx?pwid=342& |
| Initiation of Transcription | http://www.qiagen.com/products/genes%20and%20pathways/Pathway%20Details.aspx?pwid=43&resultsPerPage=50 |
| TGF_beta_Pathway | http://www.qiagen.com/products/genes%20and%20pathways/Pathway%20Details.aspx?pwid=432& |
| Transcription_of_mRNA_Pathway | http://www.qiagen.com/products/genes%20and%20pathways/Pathway%20Details.aspx?pwid=450& |
| Ubiquitin_Proteasome_Pathway | http://www.qiagen.com/products/genes%20and%20pathways/Pathway%20Details.aspx?pwid=459& |
| AKT_Pathway | http://www.qiagen.com/products/genes%20and%20pathways/Pathway%20Details.aspx?pwid=23& |
| Androgen_receptor_Pathway | http://www.qiagen.com/products/genes%20and%20pathways/Pathway%20Details.aspx?pwid=31& |
| cAMP_Pathway | http://www.qiagen.com/products/genes%20and%20pathways/Pathway%20Details.aspx?pwid=76& |
| CD40_Pathway | http://www.qiagen.com/products/genes%20and%20pathways/Pathway%20Details.aspx?pwid=90& |
| Inhibition_of_Apoptosis_Pathway | http://www.qiagen.com/products/genes%20and%20pathways/Pathway%20Details.aspx?pwid=99& |
| CREB_Pathway | http://www.qiagen.com/products/genes%20and%20pathways/Pathway%20Details.aspx?pwid=123& |
| EGFR_Pathway | http://www.qiagen.com/products/genes%20and%20pathways/Pathway%20Details.aspx?pwid=145& |
| ErbB_Family_Pathway | http://www.qiagen.com/products/genes%20and%20pathways/Pathway%20Details.aspx?pwid=159& |
| ERK_Signaling_Pathway | http://www.qiagen.com/products/genes%20and%20pathways/Pathway%20Details.aspx?pwid=162& |
| Erythropoeitin_Pathway | http://www.qiagen.com/products/genes%20and%20pathways/Pathway%20Details.aspx?pwid=165& |
| Estrogen_Pathway | http://www.qiagen.com/products/genes%20and%20pathways/Pathway%20Details.aspx?pwid=166& |
| FLT3_Signaling_Pathway | http://www.qiagen.com/products/genes%20and%20pathways/Pathway%20Details.aspx?pwid=185& |
| Growth_Hormone_Pathway | http://www.qiagen.com/products/genes%20and%20pathways/Pathway%20Details.aspx?pwid=212& |
| GSK3_Pathway | [http://www.qiagen.com/products/genes%20and%20pathways/Pathway%20Details.aspx?pwid=213](http://www.qiagen.com/products/genes and pathways/Pathway Details.aspx?pwid=213) |
| HGF_Pathway | http://www.qiagen.com/products/genes%20and%20pathways/Pathway%20Details.aspx?pwid=222& |
| HIF1Alpha_Pathway | http://www.qiagen.com/products/genes%20and%20pathways/Pathway%20Details.aspx?pwid=223& |
| IGF1R_Signaling_Pathway | http://www.qiagen.com/products/genes%20and%20pathways/Pathway%20Details.aspx?pwid=236& |
| IL_10_Pathway | http://www.qiagen.com/products/genes%20and%20pathways/Pathway%20Details.aspx?pwid=238& |
| IL_2_Pathway | http://www.qiagen.com/products/genes%20and%20pathways/Pathway%20Details.aspx?pwid=240& |
| IL_6_Pathway | http://www.qiagen.com/products/genes%20and%20pathways/Pathway%20Details.aspx?pwid=244& |
| ILK_Pathway | http://www.qiagen.com/products/genes%20and%20pathways/Pathway%20Details.aspx?pwid=246&C18 |
| Integrin_SIgnaling_Pathway | http://www.qiagen.com/products/genes%20and%20pathways/Pathway%20Details.aspx?pwid=254& |
| IP3_Pathway | http://www.qiagen.com/products/genes%20and%20pathways/Pathway%20Details.aspx?pwid=261& |
| JAK_Stat_Pathway | http://www.qiagen.com/products/genes%20and%20pathways/Pathway%20Details.aspx?pwid=263& |
| MAPK_Family_Pathway | http://www.qiagen.com/products/genes%20and%20pathways/Pathway%20Details.aspx?pwid=277& |
| MAPK_Signaling _Pathway | http://www.qiagen.com/products/genes%20and%20pathways/Pathway%20Details.aspx?pwid=282& |
| mTOR_Pathway | http://www.qiagen.com/products/genes%20and%20pathways/Pathway%20Details.aspx?pwid=304& |
| NGF_p_Pathway | http://www.qiagen.com/products/genes%20and%20pathways/Pathway%20Details.aspx?pwid=320& |
| PPAR_Pathway | http://www.qiagen.com/products/genes%20and%20pathways/Pathway%20Details.aspx?pwid=367& |
| RANK_Signaling | http://www.qiagen.com/products/genes%20and%20pathways/Pathway%20Details.aspx?pwid=379& |
| RAS_Pathway | http://www.qiagen.com/products/genes%20and%20pathways/Pathway%20Details.aspx?pwid=383& |
| Regulation_of_telomere_length_and_cellular_immortalization_effect | http://www.qiagen.com/products/genes%20and%20pathways/Pathway%20Details.aspx?pwid=43&resultsPerPage=50 |
| STAT3_Pathway | http://www.qiagen.com/products/genes%20and%20pathways/Pathway%20Details.aspx?pwid=419& |
| TNF_p_Pathway | http://www.qiagen.com/products/genes%20and%20pathways/Pathway%20Details.aspx?pwid=437& |
| TRAF_p_Pathway | http://www.qiagen.com/products/genes%20and%20pathways/Pathway%20Details.aspx?pwid=448& |
| VEGF_Pathway | http://www.qiagen.com/products/genes%20and%20pathways/Pathway%20Details.aspx?pwid=467&resultsPerPage=100 |
| Wnt_Pathway | http://www.qiagen.com/products/genes%20and%20pathways/Pathway%20Details.aspx?pwid=474& |
